# Supplementary material for: Splice-Junction-Based Mapping of Alternative Isoforms in the Human Proteome
Source: Cell Rep. Author manuscript; Available in PMC 2020 Jan 15. (PMC6961840; doi:10.1016/j.celrep.2019.11.026)
Supplement: 2 [file NIHMS1546469-supplement-2.zip › DF1/PXD010154/Lung/Lung_13_CUL1_KDTYSYLNK.pdf]

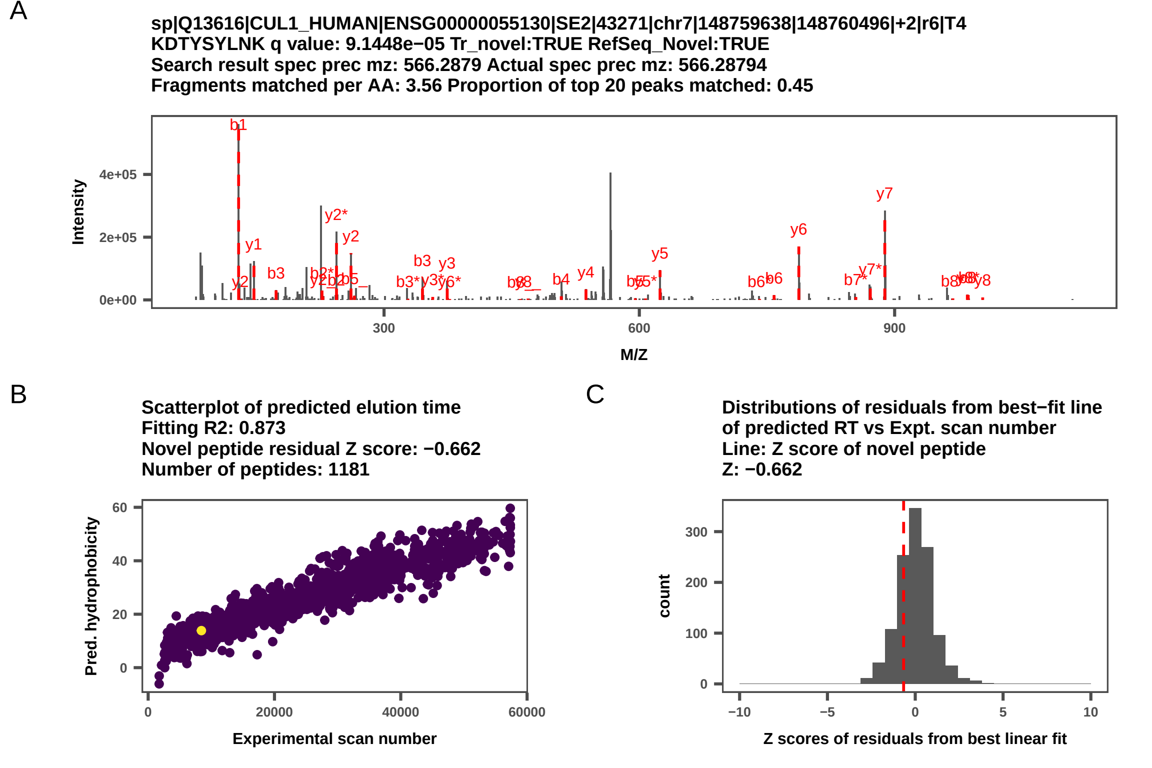
A
sp|Q13616|CUL1_HUMAN|ENSG00000055130|SE2|43271|chr7|148759638|148760496|+2|r6|T4
KDTYSYLNK q value: 9.1448e−05 Tr_novel:TRUE RefSeq_Novel:TRUE
Search result spec prec mz: 566.2879 Actual spec prec mz: 566.28794
Fragments matched per AA: 3.56 Proportion of top 20 peaks matched: 0.45
Intensity
4e+05
2e+05
0e+00
M/Z
300
600
900
b1
y1
y2
b3
y2*
y2
b2*
b5
b3*
b3
y3
y3*
y6*
b4
y4
y5
y5*
y6
b6
y7
y7*
b7*
b8
y8
B
Scatterplot of predicted elution time
Fitting R2: 0.873
Novel peptide residual Z score: −0.662
Number of peptides: 1181
Pred. hydrophobicity
Experimental scan number
0
20
40
60
0
20000
40000
60000
C
Distributions of residuals from best−fit line
of predicted RT vs Expt. scan number
Line: Z score of novel peptide
Z: −0.662
count
Z scores of residuals from best linear fit
0
100
200
300
−10
−5
0
5
10
